# Role of Serotonergic System in the Antidepressant Actions of mGlu2/3 Receptor Antagonists: Similarity to Ketamine

**DOI:** 10.3390/ijms20061270

**Published:** 2019-03-13

**Authors:** Shigeyuki Chaki, Kenichi Fukumoto

**Affiliations:** Research Headquarters, Taisho Pharmaceutical Co., Ltd., 1-403 Yoshino-cho, Kita-ku, Saitama, Saitama 331-9530, Japan; k-fukumoto@taisho.co.jp

**Keywords:** mGlu2/3 receptor antagonist, ketamine, antidepressant, serotonin, 5-HT_1A_ receptor, medial prefrontal cortex, AMPA receptor

## Abstract

Numerous studies have demonstrated the antidepressant effects of group II metabotropic glutamate (mGlu2/3) receptor antagonists in various rodent models. Importantly, it has been shown that the antidepressant effects of mGlu2/3 receptor antagonists in rodent models are similar to those of ketamine, which exerts rapid and long-lasting antidepressant effects in patients with major depressive disorders, including patients with treatment-resistant depression. In addition, the synaptic mechanisms underlying the effects of mGlu2/3 receptor antagonists are reported to be similar to those underlying the effects of ketamine. The roles of the serotonergic system in the antidepressant effects of mGlu2/3 receptor antagonists have recently been demonstrated. Moreover, it was investigated how mGlu2/3 receptor antagonists interact with the serotonergic system to exert antidepressant effects. Notably, the same neural mechanisms as those underlying the effects of ketamine may be involved in the antidepressant actions of the mGlu2/3 receptor antagonists. In this review, we shall summarize the antidepressant potential of mGlu2/3 receptor antagonists and their mechanisms of action in comparison with those of ketamine. In particular, we shall focus on the roles of the serotonergic system in the antidepressant actions of mGlu2/3 receptor antagonists.

## 1. Introduction

Glutamate is the major excitatory neurotransmitter in the brain, and is involved in numerous physiological processes in the central nervous system, including emotion and cognition. Accumulating evidence has indicated that dysfunctions of glutamatergic transmission play a key role in the etiology and pathophysiology of depression, indicating that the glutamatergic system may be a promising target for novel antidepressants [1]. At present, glutamate receptors are classified into two major types: the ionotropic glutamate (iGlu) receptors, which have an ion channel structure and comprise three subtypes, and the metabotropic glutamate (mGlu) receptors, which are coupled to G-proteins and comprise eight subtypes [2]. The importance of the glutamatergic system for developing novel antidepressants has been highlighted by the groundbreaking finding of the antidepressant effects of ketamine, a non-competitive antagonist of the *N*-methyl-D-glutamate (NMDA) receptor, one of the subtypes of the iGlu receptors. Ketamine has been demonstrated to exert rapid and robust antidepressant effects in patients with depression, including those with treatment-resistant depression (TRD), and these findings have been replicated in many institutes [3,4]. However, ketamine has serious side effects, including psychotomimetic symptoms, neurotoxicity and abuse potential, all of which preclude routine use of ketamine in daily practice. Therefore, the focus of research has now shifted to other molecular targets within the glutamatergic system, in an attempt to develop novel antidepressants devoid of ketamine-like side effects. In this context, mGlu receptors are of interest, because they exert regulatory roles on glutamatergic transmission [2]. 

mGlu receptors are classified into three groups (group I, II, and III) based on their sequence homology, second-messenger coupling, and pharmacological characteristics [2]. Of these, the group II mGlu receptors, consisting of the mGlu2 and mGlu3 receptors, are highly expressed in the cortical and limbic areas, and are negatively coupled to adenylyl cyclase activity. Therefore, mGlu2/3 receptors negatively regulate glutamate transmission in brain regions associated with emotion and cognition. mGlu2/3 receptors have been demonstrated to play critical roles in depression. Changes in the mGlu2/3 receptor expression in brain regions associated with mood were observed on postmortem examination of the brain in subjects with depression [5,6] and animal models of depression [7,8]. In addition, knockout mice lacking the mGlu2 receptor exhibited antidepressant-like phenotypes and increased reward activity [9]. In this review, we shall summarize the antidepressant potential of mGlu2/3 receptor antagonists and their mechanisms of action in comparison with those of ketamine. In particular, we shall focus on the roles of the serotonergic system in the antidepressant actions of mGlu2/3 receptor antagonists.

## 2. Antidepressant Effects of mGlu2/3 Receptor Antagonists: Similarity to Ketamine

To date, several laboratories have demonstrated and characterized the antidepressant effects of mGlu2/3 receptor antagonists in a variety of animal models, mostly by using the orthosteric mGlu2/3 receptor antagonists MGS0039, LY341495, and LY3020371 (see review, [10]). Notably, these studies have shown that mGlu2/3 receptor antagonists exert the antidepressant effects similar to those of ketamine in rodent models. For example, MGS0039 and LY341495 exerted antidepressant effects within 24 h of administration in a chronic social defeat stress model [11] and chronic unpredictable stress model [12], similar to ketamine [11,13]. In these models, the currently available antidepressants take a few weeks to exert their antidepressant effects. Furthermore, the antidepressant effects of mGlu2/3 receptor antagonists and ketamine in these models lasted for a week after a single administration [11,12,13], suggesting that the mGlu2/3 receptor antagonists may also have ketamine-like rapid and sustained antidepressant effects. Moreover, mGlu2/3 receptor antagonists also exerted antidepressant effects in some animal models that are relatively resistant to currently available antidepressants, such as corticosterone-treated rodents and CD1 mice [14,15,16]. Similarly, ketamine, which has been demonstrated to show potent antidepressant effects in patients with TRD, also showed efficacy in these models. Therefore, mGlu2/3 receptor antagonists are expected to exert antidepressant effects even in patients who show inadequate response to currently available antidepressant therapies. Importantly, unlike ketamine, mGlu2/3 receptor antagonists have not been shown to increase locomotor activity, impair cognition, or induce abuse potential [17,18]. From this perspective, mGlu2/3 receptor antagonists can be novel antidepressant drugs with similar efficacy to ketamine, but devoid of the unwanted effects of ketamine.

The roles of individual subtypes of mGlu receptors (mGlu2 receptor and mGlu3 receptor) in the antidepressant actions of the mGlu2/3 receptor antagonists are still unclear. LY341495 no longer exerted antidepressant effects in knockout mice lacking the mGlu2 receptor, but its effects were still observed in mice lacking the mGlu3 receptor [19], suggesting that mGlu2, and not mGlu3, may have a more critical role in the actions of the mGlu2/3 receptor antagonists. On the other hand, recently, selective mGlu3 receptor-negative allosteric modulators (NAMs) and selective mGlu2 receptor NAMs, both of which bind to sites of the receptor different from the glutamate-binding site, have been developed, and recent studies using these selective mGlu2 receptor NAMs and mGlu3 receptor NAMs yielded different results from those described above. According to one study, a selective mGlu3 receptor NAM exerted antidepressant effects in the tail suspension test (TST) comparable to those of ketamine, whereas a selective mGlu2 receptor NAM did not show any significant antidepressant effects [20], suggesting that mGlu3 receptor blockade may play a greater role in the antidepressant effects of mGlu2/3 receptor antagonists.

mGlu2/3 receptor antagonists have been reported to share not only the antidepressant profile of ketamine, but also the mechanisms underlying the antidepressant effects. It has been proposed that ketamine indirectly stimulates the α-amino-3-hydroxy-5-methyl-isoxazole-4-propionate (AMPA) receptor via disinhibition of GABA interneurons, thereby, increasing brain-derived neurotrophic factor (BDNF) secretion and synthesis, which subsequently activates mechanistic target of rapamycin complex 1 (mTORC1) signaling in particular brain regions, such as the medial prefrontal cortex (mPFC) [21]. These events eventually increase synaptic protein synthesis, leading to increased spine density in the regions [21]. It is of interest that chronic stress reduced synaptic protein synthesis, spine density, and synaptic functions, and ketamine rapidly reversed these synaptic abnormalities, even after a single administration [13]. Stimulations of the AMPA receptor, BDNF/tropomyosin-related kinase B (TrkB) signaling and mTORC1 signaling have also been reported to mediate the antidepressant effects of mGlu2/3 receptor antagonists [22,23,24,25]. Moreover, the mGlu2/3 receptor antagonist also reversed chronic stress-induced decrease of synaptic protein synthesis and spine density in the mPFC [11]. Therefore, normalization of the synaptic functions in the mPFC disrupted by chronic stress may have a critical role in the antidepressant effects of both mGlu2/3 receptor antagonists and ketamine.

## 3. Role of the Serotonergic System in the Antidepressant Effects of mGlu2/3 Receptor Antagonists and Ketamine

Serotonergic transmission has long been known to play critical roles in the actions of various antidepressants, and has been implicated in the etiology and pathophysiology of depression (see review, [26]). It has been reported that the mGlu2/3 receptor antagonists and ketamine also exert effects on the serotonergic system. We previously reported that mGlu2/3 receptor antagonists increased the firing of the serotonin (5-HT) neurons in the dorsal raphe nucleus (DRN) and increased the extracellular 5-HT levels in the rat mPFC [27], and that increased 5-HT release by the mGlu2/3 receptor antagonist was attenuated by an AMPA receptor antagonist [28]. Therefore, mGlu2/3 receptor antagonists activate the serotonergic system through activation of the AMPA receptor. Ketamine has also been reported to increase the extracellular 5-HT levels in the mPFC in mice, mediated by AMPA receptor stimulation [29,30]. Furthermore, in the novelty-suppressed feeding test (NSFT) and forced swimming test (FST), the antidepressant actions of LY341495 and ketamine were blocked by pretreatment with para-chlorophenylalanine (PCPA), an irreversible inhibitor of tryptophan hydroxylase, which caused sufficient pharmacological depletion of 5-HT in the brain (more than 70% reduction of the cortical 5-HT content), suggesting that the serotonergic system in the brain plays essential roles in the antidepressant effects of both the mGlu2/3 receptor antagonist and ketamine [30,31,32,33]. Collectively, these findings suggest that AMPA receptor-dependent 5-HT release in the mPFC may be closely involved in the antidepressant effects of the mGlu2/3 receptor antagonist and ketamine.

Accumulated evidence indicates that among the 14 known 5-HT receptor subtypes, the 5-HT_1A_ receptor is the most closely related to the antidepressant effects of drugs [26]. The antidepressant effects of the mGlu2/3 receptor antagonist and ketamine have also been reported to be blocked by a 5-HT_1A_ receptor antagonist, but not by a 5-HT_2A/2C_ receptor antagonist, indicating that activation of the 5-HT_1A_ receptors is involved in the antidepressant effects of the mGlu2/3 receptor antagonist and ketamine [31,34,35]. Although there is less evidence as compared to that for the involvement of the 5-HT_1A_ receptor, other 5-HT receptor subtypes and the 5-HT transporter (SERT) in the antidepressant effects of ketamine has also been reported. In a positron emission tomography (PET) study in non-human primates, ketamine was shown to increase the binding to 5-HT_1B_ receptor in the nucleus accumbens and ventral pallidum of rhesus monkeys, mediated by activation of the AMPA receptor. Thus, upregulation of 5-HT_1B_ receptors in the nucleus accumbens and ventral pallidum may be involved in the antidepressant effects of ketamine [36]. Role of the 5-HT_1B_ receptor was also reported in a study showing that the antidepressant effect of (S)-ketamine, the S(+) enantiomer of ketamine, required the activation of the 5-HT_1B_ receptor in 5-HT-depleted Flinders sensitive line rats, a genetic model of depression [37]. Moreover, treatment with an antidepressant dose (1.5 mg/kg over 40 min) of ketamine was reported to increase the 5-HT release in the PFC of rhesus monkeys, presumably through reduction of SERT, indicating that ketamine enhanced serotonergic transmission by inhibiting the SERT activity [38]. Given that ketamine does not bind to SERT at concentrations observed after administration of antidepressant doses in humans [39], ketamine may indirectly reduce binding of the PET tracer. Ketamine has been reported to upregulate the expression of the 5-HT_2C_ receptor mRNA and microRNA (miRNA) cluster in the mouse hippocampus via the AMPA receptor and glycogen synthase kinase-3, and the antidepressant effects of ketamine in a learned helplessness model were shown to be attenuated by an antagonist to miRNA 448-3p (one of the 5-HT_2C_ receptor miRNAs), suggesting the involvement of the 5-HT_2C_ receptor in the antidepressant effects of ketamine [40]. These findings also strongly support the involvement of the serotonergic system in the actions of ketamine, while the roles of each of the 5-HT receptor subtypes need to be investigated further. Likewise, studies on the roles of the 5-HT receptor subtypes other than the 5-HT_1A_ receptor in the antidepressant actions of mGlu2/3 receptor antagonists need to be further investigated. The recognized roles of each of the 5-HT receptor subtypes in the antidepressant effects of the mGlu2/3 receptor antagonists and ketamine are summarized in Table 1. In contrast, opposite findings that the antidepressant effects of the mGlu2/3 receptor antagonist and ketamine are independent of the serotonergic system have also been reported. The antidepressant effects of MGS0039, an mGlu2/3 receptor antagonist, in TST were not blocked by either 5-HT depletion or blockade of the 5-HT_1A_ receptor and 5-HT_2A/2C_ receptor, suggesting the unlikely involvement of the serotonergic system in the antidepressant effects of the mGlu2/3 receptor antagonists [41]. In addition, it has also been reported that 5-HT depletion had no effects on the antidepressant effects of (R)-ketamine, an R(-) enantiomer of ketamine, in a chronic social defeat stress model, indicating that the unlikely involvement of the serotonergic system in the actions of (R)-ketamine [42]. Although these differences may be ascribed to differences in the experimental conditions or animal models used among the studies, the precise reason remains unclear at present.

## 4. Mechanisms of Interaction between the Serotonergic and Glutamatergic Systems

**Figure 1:**
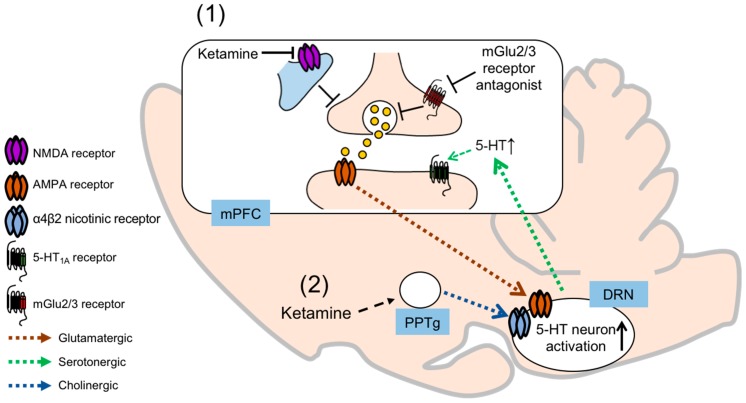
Proposed serotonergic mechanisms through which mGlu2/3 receptor antagonists and ketamine exert their antidepressant effects. Both mGlu2/3 receptor antagonists and ketamine increase 5-HT release in the medial prefrontal cortex (mPFC) through activation of 5-HT neurons in the dorsal raphe nucleus (DRN). There are two hypotheses by which mGlu2/3 receptor antagonists and ketamine activate 5-HT neurons in the DRN. (1) mGlu2/3 receptor antagonists and ketamine activate neurons in the mPFC projecting to 5-HT neurons in the DRN. Stimulation of AMPA receptor both in the mPFC (to activate neurons projecting to the DRN) and in the DRN (to activate 5-HT neurons) may be involved in this pathway. mGlu2/3 receptor antagonists and ketamine indirectly stimulate AMPA receptor by increasing glutamate release in different manners. (2) Ketamine activates cholinergic neurons in the pedunculopontine tegmental nucleus (PPTg), projecting to the DRN neurons where α4β2 nicotinic receptor, together with AMPA receptor, activates 5-HT neurons. Activation of 5-HT neurons in the DRN leads to increase in 5-HT release in the mPFC, and stimulates postsynaptic 5-HT_1A_ receptor.

To date, the precise mechanisms of how mGlu2/3 receptor antagonists and ketamine affect serotonergic transmission remain to be fully understood. Recent studies have suggested roles of the mPFC-DRN pathway in the regulation of the serotonergic system by both compounds, and also the role of this pathway in the antidepressant effects of the compounds. Injection of LY341495 or ketamine into the mPFC has recently been reported to increase the number of c-Fos-positive 5-HT neurons in the DRN, and the increase in the number of c-Fos positive 5-HT neurons in the DRN induced by systemic administration of LY341495 or ketamine was blocked by intra-mPFC injection of an AMPA receptor antagonist [32]. Thus, mGlu2/3 receptor antagonists and ketamine stimulate the AMPA receptor in the mPFC, which leads to an increase in activity of 5-HT neurons in the DRN, presumably via the mPFC-DRN projection. Because the antidepressant effects of LY341495 and ketamine were attenuated by intra-mPFC injection of an AMPA receptor antagonist, it has been concluded that mPFC AMPA receptor stimulation is necessary for mGlu2/3 receptor antagonists and ketamine to exert their antidepressant effects. Considering these results, together with the result that the antidepressant effects of intra-mPFC injection of LY341495 or ketamine were abrogated by depletion of endogenous 5-HT [32], it is conceivable that mPFC AMPA receptor stimulation-induced 5-HT neuron activation in the DRN plays a critical role in the antidepressant effects exerted by LY341495 and ketamine. Pham et al. [30,43] reported that intra-mPFC injection of ketamine increased 5-HT release in the mPFC, which coincided with the antidepressant effects in the FST, and intra-DRN injection of an AMPA receptor antagonist blocked these effects of ketamine. Therefore, increase of mPFC 5-HT release via stimulation of the AMPA receptor in the DRN through the mPFC-DRN projection may be involved in the antidepressant effects of ketamine [30,43]. Although the study by Pham et al. was restricted to the actions of ketamine, the observations that mGlu2/3 receptor antagonists increase 5-HT release in the mPFC via AMPA receptor stimulation [28] and that the antidepressant effects of LY341495 were blocked by silencing of the neuronal activity in the DRN (by local injection of muscimol into the DRN) [34] clearly indicate that DRN 5-HT activation, and the consequent increase in 5-HT release in the mPFC, are also critical for the antidepressant effects exerted by mGlu2/3 receptor antagonists. The importance of the mPFC-DRN projection in the antidepressant effects of these compounds has also been demonstrated by other studies. It was reported that stimulation of the AMPA receptor and neural activity in the infralimbic cortex (a ventral subdivision of the mPFC) by local injection of S-AMPA or transient GLT-1 blockade (increase in extracellular glutamate) increased extracellular 5-HT expression and number of c-Fos positive 5-HT cells in the DRN, coinciding with the antidepressant effects [44,45]. This is another evidence suggesting that mPFC AMPA receptor stimulation activates the mPFC-DRN projection, which in turn activates the 5-HT neurons in the DRN, and that these events are associated with antidepressant effects. Notably, the antidepressant effects induced by injection of a GLT-1 inhibitor into the infralimbic cortex were attenuated by 5-HT depletion and the infralimbic cortex injection of the AMPA receptor antagonist. Therefore, activation of 5-HT neurons via the mPFC-DRN pathway triggered by AMPA receptor stimulation in the mPFC (and possible subsequent 5-HT release in the mPFC) may have crucial roles in the antidepressant effects. It should be noted, however, that the antidepressant effects of GLT-1 blockade or AMPA receptor stimulation in the infralimbic cortex are not sustained, which is distinct from the antidepressant actions of mGlu2/3 receptor antagonists and ketamine. The role of the mPFC-DRN projection in the antidepressant effects of mGlu2/3 receptor antagonists and ketamine is underscored by previous reports showing that optogenetic stimulation of mPFC cells projecting to the DRN was associated with robust antidepressant effects [46], and that optogenetic stimulation of the infralimbic cortex mimicked the antidepressant profiles of ketamine [47]. On the other hand, Nishitani et al. proposed a different mechanism underlying the stimulation by ketamine of the DRN 5-HT neurons to increase 5-HT release in the PFC. They reported that ketamine increases 5-HT release in the PFC via stimulation of the AMPA receptors and α4β2 nicotinic receptors in the DRN, since the ketamine-induced 5-HT release in the PFC was blocked by local injection of an AMPA receptor antagonist or an α4β2 nicotinic receptor antagonist into the DRN [29]. In their study, local injection of ketamine into the DRN did not increase 5-HT release in the PFC, which led them to indicate that ketamine may not directly activate the 5-HT neurons in the DRN, but rather indirectly induce 5-HT release via pathways projecting to the DRN. Moreover, they reported that ketamine acted on the pedunculopontine tegmental nucleus to activate cholinergic neurons projecting to the DRN, which eventually stimulate both the AMPA receptors and α4β2 nicotinic receptors in the DRN to stimulate 5-HT release in the PFC [48]. Given that acetylcholine activates 5-HT neurons by increasing glutamate release through presynaptic α4β2 nicotinic receptors in the DRN [49], it has been hypothesized that the ketamine-activated cholinergic projection to the DRN indirectly stimulates the AMPA receptors in the region. Thus, further investigation is needed for elucidation of the precise mechanisms by which ketamine activates the DRN 5-HT neurons, which project to the mPFC to increase 5-HT release. In addition, it is necessary to demonstrate if mGlu2/3 receptor antagonists and ketamine share the same mechanisms of actions. The proposed serotonergic mechanisms through which mGlu2/3 receptor antagonists and ketamine exert their antidepressant effects are illustrated in Figure 1.

It should be mentioned here that there is at least one study that reported that ketamine did not affect the DRN 5-HT neurons. El Iskandrani et al. [50] reported that systemic administration of ketamine increased the firing activity of norepinephrine (NE) neurons in the locus coeruleus (LC) through AMPA receptor stimulation, while exerting no such effect on the DRN 5-HT neurons or ventral tegmental area (VTA) dopamine neurons. In contrast, we previously reported that systemic administration of mGlu2/3 receptor antagonists increased the firing rate of the DRN 5-HT neurons, while it had no effect on the firing rate of the LC NE neurons [27]. Witkin et al. [16] reported that both mGlu2/3 receptor antagonists and ketamine increased the number of spontaneously active dopamine neurons in the VTA.

## 5. Mechanisms of Interaction between the Serotonergic System and Synaptic Plasticity 

As we have mentioned above, mGlu2/3 receptor antagonists and ketamine share some of the mechanisms underlying their antidepressant effects, including involvement of the serotonergic system and synaptic neuroplasticity. However, it is not exactly known how the serotonergic system and synaptic neuroplasticity are related. It has been reported that the sustained antidepressant effects of the mGlu2/3 receptor antagonist and ketamine were blocked by selective blockade of the 5-HT_1A_ receptor in the mPFC [34,35]. Importantly, infusion of a 5-HT_1A_ receptor agonist into the mPFC induced sustained antidepressant effects, similar to infusion of an mGlu2/3 receptor antagonist or ketamine, while systemic administration of a 5-HT_1A_ receptor agonist showed no such effects. 5-HT_1A_ receptors are known to exist both presynaptically and postsynaptically, and the 5-HT_1A_ receptors are expressed as postsynaptic receptors in the mPFC. Therefore, these findings suggest that the selective stimulation of the postsynaptic 5-HT_1A_ receptor in the mPFC plays an important role in the sustained antidepressant effects of both mGlu2/3 receptor antagonist and ketamine. The imaging and postmortem studies showed the importance of the mPFC in the pathophysiology and treatment of depression, supporting this suggestion [51,52]. 

**Figure 2:**
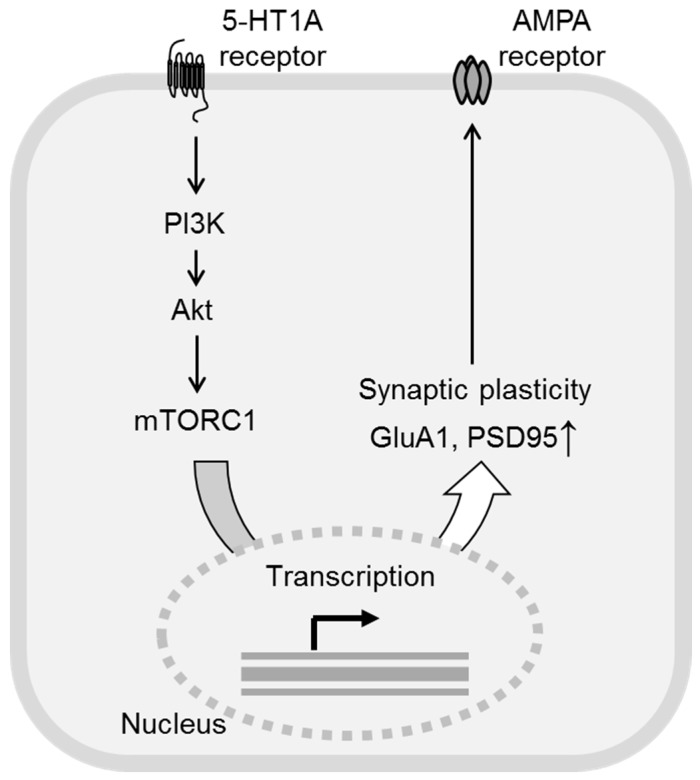
Proposed 5-HT_1A_ receptor-mediated signaling pathway through which mGlu2/3 receptor antagonists and ketamine exert their antidepressant effects. 5-HT release induced by mGlu2/3 receptor antagonists and ketamine as described in Figure 1 stimulates postsynaptic 5-HT_1A_ receptor in the medial prefrontal cortex (mPFC). 5-HT_1A_ receptor stimulation activates phosphoinositide-3-kinase (PI3K) and subsequently Akt, leading to activation of mechanistic target of rapamycin complex 1 (mTORC1) signaling. mTORC1 signaling increased synthesis of synaptic proteins such as GluA1 and PSD95, which induces synaptic plasticity. Increase in GluA1 synthesis persistently activates AMPA receptor transmission, which may also be involved in sustained antidepressant effects of mGlu2/3 receptor antagonists and ketamine.

Although the sustained antidepressant effects of mGlu2/3 receptor antagonists, such as LY341495 and MGS0039, have been reported even at 24 h and 1 week after the drug administration [11,12], the elimination half-lives of LY341495 (10 mg/kg, i.p.) and MGS0039 (3 mg/kg, p.o.) in the brain are only approximately 4.75 h and 2.15 h, respectively, similar to the elimination half-life of ketamine. Therefore, the sustained antidepressant effects of mGlu2/3 receptor antagonists are exerted via induction of synaptic plasticity [11,23,53,54,55]. Ketamine has been reported to induce sustained antidepressant effects through the activation of phosphoinositide 3-kinase (PI3K)/Akt signaling and subsequent induction of synaptic plasticity [13]. The PI3K/Akt signaling pathway is known as one of the downstream signaling pathways of the 5-HT_1A_ receptor, because the phosphorylation of Akt induced by 8-OH-DPAT, a 5-HT_1A_ receptor agonist, was blocked by LY294002, an inhibitor of PI3K [56]. Moreover, activation of the 5-HT_1A_ receptor is reported to be involved in the synaptic plasticity via activation of Akt [57]. Therefore, it is possible that stimulation of the 5-HT_1A_ receptors activates the PI3K/Akt signaling pathway and consequently induces synaptic plasticity. It has recently been demonstrated that the sustained antidepressant effects of the mGlu2/3 receptor antagonist and ketamine were blocked by infusion of a PI3K inhibitor into the mPFC. It was also shown that an mGlu2/3 receptor antagonist and ketamine increased the phosphorylation of Akt in the mPFC and that these effects were blocked by infusion of a 5-HT_1A_ receptor antagonist or PI3K inhibitor into the mPFC. These findings suggest that mGlu2/3 receptor antagonists and ketamine activate the PI3K/Akt pathway via stimulation of the 5-HT_1A_ receptor in the mPFC, and that this mechanism is responsible, at least in part, for the sustained antidepressant effects exerted by these drugs. This hypothesis is underpinned by the finding that the sustained antidepressant effects exerted by infusion of a 5-HT_1A_ receptor agonist into the mPFC were blocked by intra-mPFC infusion of a 5-HT_1A_ receptor antagonist or a PI3K inhibitor [35]. PI3K/Akt signaling is also known to activate mTORC1 signaling to induce synaptic plasticity [58], and mTORC1 signaling has been shown to be involved in the sustained antidepressant effects of mGlu2/3 receptor antagonists and ketamine [13,22,23]. Interestingly, it was demonstrated that the sustained antidepressant effects of an mGlu2/3 receptor antagonist and ketamine were blocked by infusion of an mTORC1 inhibitor into the mPFC, suggesting that the sustained antidepressant effects of mGlu2/3 receptor antagonists and ketamine are mediated via mTORC1 signaling [34,35]. These findings are consistent with the findings of a previous study that showed that intracerebroventricular injection of an mTORC1 inhibitor blocked the sustained antidepressant effects of an mGlu2/3 receptor antagonist and ketamine [13,22,23]. Collectively, all these findings indicate that mGlu2/3 receptor antagonists and ketamine activate the PI3K/Akt/mTORC1 signaling pathway via the stimulation of the 5-HT_1A_ receptors in the mPFC and induce synaptic plasticity to exert sustained antidepressant effects. Notably, the sustained antidepressant effects of infusion of a 5-HT_1A_ receptor agonist into the mPFC was also blocked by infusion of an mTORC1 inhibitor into the mPFC [35], suggesting that the mPFC 5-HT_1A_ receptor-mediated PI3K/Akt stimulation leads to activation of mTORC1 signaling, resulting in sustained antidepressant effects (Figure 2). Moreover, this finding also implies the importance of selective stimulation of 5-HT_1A_ receptors in the mPFC as a new target for the treatment of depression, because this mechanism is also shared by ketamine. The importance of selective stimulation of the 5-HT_1A_ receptor in the mPFC is also supported by the finding that F15599, which preferentially activates the postsynaptic 5-HT_1A_ receptors, induced more potent and sustained antidepressant effects than F13714, which acts on both the postsynaptic and presynaptic 5-HT_1A_ receptors [59].

## 6. Conclusions and Future Directions

As described above, accumulating evidence has clearly indicated that serotonergic transmission plays critical roles in the antidepressant effects of both mGlu2/3 receptor antagonists and ketamine. Moreover, activation of the DRN 5-HT neurons via selective stimulation of the mPFC-DRN pathway and subsequent stimulation of the 5-HT_1A_ receptors in the mPFC (presumably via increased 5-HT release in the mPFC) are involved in the sustained antidepressant effects of both mGlu2/3 receptor antagonists and ketamine. This assumption is underpinned by the results that selective stimulation of the mPFC 5-HT_1A_ receptors mimics not only the sustained antidepressant effects of the mGlu2/3 receptors, but also the roles of the signaling mechanisms (PI3K/AKT/mTORC1) that are also required for mGlu2/3 receptor antagonists and ketamine to exert their sustained antidepressant effects. Therefore, the findings of investigations conducted to elucidate the neural and synaptic mechanisms underlying the antidepressant actions of mGlu2/3 receptor antagonists and ketamine led to the hypothesis that selective stimulation of the mPFC 5-HT_1A_ receptor is an attractive target for developing novel ketamine-like antidepressants. However, there are still some issues that need to be resolved: (1) Does stimulation of the mPFC 5-HT_1A_ receptors increase synaptic protein synthesis and spine formation? (2) How do mGlu2/3 receptor antagonists and ketamine selectively stimulate the 5-HT_1A_ receptors in the mPFC? (3) Do postsynaptic 5-HT_1A_ receptors in other brain regions have roles in the antidepressant effects of mGlu2/3 receptor antagonists and ketamine? Eventually, the above mentioned hypothesis needs to be proven in clinical studies using agents that selectively activate the mPFC 5-HT_1A_ receptor. Moreover, role of each mGlu receptor subtypes, mGlu2 receptor and mGlu3 receptor, in the actions of mGlu2/3 receptor antagonists on serotonergic system needs to be elucidated. The serotonergic system has long been considered to have critical roles in the antidepressant effects. Further investigation of the roles of the serotonergic system in the antidepressant effects of mGlu2/3 receptor antagonists and ketamine should open an exciting avenue for the development of safer and more efficacious antidepressants.

## Figures and Tables

**Table 1:** Summary of role of serotonergic system in antidepressant effects of mGlu2/3 receptor antagonists and ketamine.

Agents		Animals	Effects	Role of Serotonergic System	References
**mGlu2/3 receptor** **antagonists**	LY341495 (i.p., 30 min after the treatment)	Mice	Antidepressant effects (NSFT) was blocked by PCPA (i.p.) and WAY100635 (s.c.) Antidepressant effects (NSFT) was NOT blocked by ritanserin (i.p.)	1. Serotonergic system is involved in acute antidepressant effect 2. 5-HT_1A_ receptor stimulation is involved in acute antidepressant effect 3. 5-HT_2A/2C_ stimulation is NOT involved in acute antidepressant effect	[31]
	LY341495 (i.p., 30 min or 24 h after the treatment)	Mice	Antidepressant effects (FST) was blocked by PCPA (i.p.)	1. Serotonergic system is involved in acute and sustained antidepressant effect	[32]
	LY341495 (i.p. 30 min or 24 h after the treatment)	Mice	Antidepressant effects (FST) was blocked by WAT100635 (i.p.) and WAY100635 (intra-mPFC)	1. 5-HT_1A_ receptor stimulation in the mPFC is involved in acute and sustained antidepressant effect	[34]
	MGS0039 (i.p., 30 min after the treatment)	Mice	Antidepressant effects (TST) was NOT blocked by PCPA (i.p.), WAY100635 (s.c.) or ritanserin (i.p.)	1. Serotonergic system is NOT involved in acute antidepressant effects 2. 5-HT_1A_ receptor stimulation is NOT involved in acute antidepressant effect 3. 5-HT_2A/2C_ stimulation is NOT involved in acute antidepressant effect	[41]
	MGS0039 (i.p., 3 h postinjection sample collection)	Rats	Increase of extracellular 5-HT conc in the mPFC (microdialysis) was blocked by NBQX (s.c.)	1. AMPA receptor stimulation is involved in increase of 5-HT release in the mPFC	[28]
	MGS0039 (i.p. or i.v., 2 h postinjection sample collection)	Rats	Increase of extracellular 5-HT conc in the mPFC (microdialysis) Activation of firing of 5-HT neuron in the DRN (single cell recording)	1. Increase of 5-HT release in the mPFC 2. Activation of 5-HT neurons in the DRN	[27]
Ketamine	Ketamine (i.p., 30 min after the treatment)	Mice	Antidepressant effects (NSFT) was blocked by PCPA (i.p.) and WAY100635 (s.c.) Antidepressant effects (NSFT) was NOT blocked by ritanserin (i.p.)	1. Serotonergic system is involved in acute antidepressant effects 2. 5-HT_1A_ receptor stimulation is involved in acute antidepressant effect 3. 5-HT_2A/2C_ stimulation is NOT involved in acute antidepressant effect	[31]
	Ketamine (i.p., 30 min or 24 h after the treatment)	Mice	Antidepressant effects (FST) was blocked by PCPA (i.p.)	1. Serotonergic system is involved in acute and sustained antidepressant effect	[32]
	Ketamine (i.p., 30 min or 24 h after the treatment)	Mice	Antidepressant effects (FST) was blocked by WAT100635 (i.p.) and WAY100635 (intra-mPFC)	1. 5-HT_1A_ receptor stimulation in the mPFC is involved in acute and sustained antidepressant effect	[35]
	Ketamine (i.p., 24 h after the treatment)	Rats	Antidepressant effects (FST) was blocked by PCPA (i.p.)	1. Serotonergic system is involved in sustained antidepressant effect	[33]
	Ketamine (i.p., 24 h after the treatment)	Mice	Antidepressant effects (FST) was blocked by PCPA (i.p.) and NBQX (intra-DRN)	1. Serotonergic system is involved in sustained antidepressant effect 2. AMPA receptor stimulation in the DRN is involved in sustained antidepressant effect	[43]
			Increase of extracellular 5-HT conc in the mPFC (microdialysis) was blocked by NBQX (intra-DRN)	1. Increase of 5-HT release in the mPFC 2. AMPA receptor activation in the DRN is involved in 5-HT release in the mPFC	
	Ketamine (i.p., 24 h after the treatment)	Mice	Increase of extracellular 5-HT conc in the cortex (microdialysis)	1. Increase of 5-HT release in the cortex	[30]
	Ketamine (i.p., 24 h after the treatment)	Mice	Increase of extracellular 5-HT conc in the mPFC (microdialysis) was blocked by NBQX (intra-DRN)	1. AMPA receptor stimulation in the DRN is involved in increase of 5-HT release in the mPFC	[29]
	(S)-Ketamine (i.p., 1 h or 48 h after the treatment)	5-HT depleted Flinders sensitive line rats	Antidepressant effects (FST) was blocked by PCPA (i.p.) CP04253 (i.p.) restored antidepressant effect (FST) of (S)-ketamine in PCPA-treated animals	1. Serotonergic system is involved in acute and sustained antidepressant effect 2. Stimulation of 5-HT_1B_ receptor is involved in acute and sustained antidepressant effect	[37]
	Ketamine (i.v., 100 min after the bolus treatment and continuous treatment)	Rhesus monkeys	Increase of [^11^C]AZ10419369 binding in the nucleus accumbens and ventral pallidum (PET) Increase of [^11^C]AZ10419369 binding (PET) was blocked by NBQX (i.v.)	1. Increase of 5-HT_1B_ receptor binding in the nucleus accumbens and ventral pallidum 2. AMPA receptor stimulation is involved in increase of 5-HT_1B_ receptor binding	[36]
			Decrease of [^11^C]DASB binding in the nucleus accumbens and ventral pallidum (PET) Decrease of [^11^C]DASB binding (PET) was not blocked by NBQX (i.v.)	1. Decrease of 5-HT transporter binding in the nucleus accumbens and ventral pallidum 2. AMPA receptor stimulation is NOT involved in decrease of 5-HT transporter binding	
	Ketamine (i.v., 40 min after the treatment)	Rhesus monkeys	Decrease of [^11^C]DASB binding in the midbrain, thalamus, striatum, PFC (PET) Increase of extracellular 5-HT conc but not extracellular dopamine conc in the PFC (microdialysis) No change in [^18^F]MPPF binding and [^11^C]b-CFT binding (PET)	1. Decrease of 5-HT transporter binding in global resions 2. Increase of 5-HT release but NOT dopamine release in the PFC 3. No change of 5-HT_1A_ receptor binding and dopamine transporter binding	[38]
	Ketamine (i.p., 24 h after the treatment)	Mice	Increase of 5-HT_2C_ mRNA and miRNA cluster in the hipocampus Increase of 5-HT_2C_ mRNA and miRNA cluster were blocked by GSK3b knock-in and NBQX (i.p.)	1. Up regulation of 5-HT_2C_ receptor expression 2. AMPA receptor stimulation and GSK3b inhibition are involved in up regulation of 5-HT_2C_ receptor expression	[40]
			Antidepressant effects (LH) was blocked by miRNA 448-3p (i.p.)	1. 5-HT_2C_ receptor up regulation is involved in sustained antidepressant effect	
	(R)-Ketamine (i.p., 1 h or 48 h after the treatment)	Mice	Antidepressant effects (CSDS, TST, SPT) was NOT blocked by PCPA (i.p.)	1. Serotonergic system is NOT involved in acute and sustained antidepressant effect	[42]
	Ketamine (i.v., 40 min after the treatment)	Human	No change of [^11^C]DASB binding in the caudate, putamen, thalamus (PET)	1. No change of 5-HT transporter binding in the the caudate, putamen, thalamus	[39]

## Abbreviation

iGlu	ionotropic glutamate
mGlu	metabotropic glutamate
NAM	negative allosteric modulator
TST	tail suspension test
FST	forced swimming test
NSFT	novelty-suppressed feeding test
SERT	5-HT transporter
AMPA	α-amino-3-hydroxy-5-methyl-isoxazole-4-propionate
BDNF	brain-derived neurotrophic factor
mTORC1	mechanistic target of rapamycin complex 1
TrkB	tropomyosin-related kinase B
PI3K	phosphoinositide 3-kinase
miRNA	microRNA
mPFC	medial prefrontal cortex
DRN	dorsal raphe nucleus
PET	positron emission tomography
PPTg	pedunculopontine tegmental nucleus
LC	locus coeruleus
VTA	ventral tegmental area
5-HT	serotonin
